# Society for the Study of Comparative Psychology in Connection with the Faculty of Comparative Medicine and Veterinary Science of McGill University, Montreal, Canada

**Published:** 1893-12

**Authors:** 


### Report of the Proceedings of the Society for the Study of Comparative Psychology in Connection with the Faculty of Comparative Medicine and Veterinary Science of McGill University, Montreal, Canada, for the Session of 1892–93.

A meeting of the Society was held at the usual place, the lecture-room of the Faculty of Comparative Medicine and Veterinary Science, for the election of officers, on Nov. 2nd. 1893, with the following results: Hon. Pres., Dr. D. McEachran; President, Dr. Wesley Mills; 1st Vice-Pres., Dr. M. C. Baker; 2nd Vice-Pres., Mr. Brainerd; Sec.-Treas., Mr. Sturroch; Corresponding Sec., Mr. Ewing.

A meeting was held on Nov. 14th, the president, Dr. Mills, occupying the chair. The attendance was large, and after the minutes of the preceding meeting had been read the question was raised as to the advisability of offering prizes for essays of sufficient originality and merit. Several gentlemen present expressed their views on the subject, and it was finally decided by vote of the majority that two prizes should be offered for the two best essays read this session.

Mr. Glen Campbell read an interesting paper, entitled "The Child and Our Domestic Pets," and Mr. A. S. Cleaves one on "Animal Psychology." The gist of the former gentleman's thesis was a comparison of the psychic development of the child, the colt, and the dog. He pointed out that those animals that are produced singly seem to be the best, that "the noblest are ever the least fruitful" The dog, however, was an exception, probably due to man's interference by artificial selection, by education and by domestication. The modes of communication, one with the other, amongst the lower animals are somewhat difficult to understand. Our own language was crude in certain respects, taking for example the absence of a full language for smell. On the other hand, the sense of smell in the dog was most acute, the great memory for smell being a prominent characteristic of some breeds. By the association of impressions we draw conclusions, as when we determine what a substance is by seeing and then feeling it, or by tasting and smelling it, and in like manner the dog recognizes his master by the senses of sight and smell. Dogs are known to express their dislike of one another by the erection of the hair, and often, after having been romped with and been teased by their masters for a considerable time, will express their desire for a rest by growling. This is their natural means of asking to be left in peace, and why should their wishes not be complied with? He mentioned that at the present day the question of "mental telegraphy" was engrossing the minds of many scientists. In France and other continental countries it is customary for the regimental veterinary surgeon to inspect the horses daily before going to drill. Some of these horses actually knew when the doctor was on his rounds, and would suddenly become lame and feign sickness in order to escape being sent out to work. Love of the young is seen in all animals. He instanced a case of a dog carrying her puppies off the track on hearing and seeing an approaching train. This animal must have anticipated danger. Unfortunately, some time after the dog was itself run over and killed. Mr. Cleaves advised the members of the society never to neglect an opportunity of pointing out the existence of reasoning power amongst our dumb friends to any incredulous persons with whom they might come in contact. He believed, with most writers, that intelligence had reached a higher state of development in pure-bred animals than in mongrels. He related how a horse, which had been over the way before, had led its owner safely out of a wood in which he had got lost. Another horse he owned at home appeared to distinguish between the harness used for driving and that for work. He suggested that much of the suffering of the human inmates of our hospitals was in great measure the result of their own folly, whilst that of the poor horses in our cities was frequently due to the brutality and thoughtlessness of their owners. Legislation, as a preventive means, did not seem efficient. Example and persuasion appeared to have more influence.

The reading of the papers was followed by a short discussion. Dr. Mills said he thought Mr. Campbell's statement about the dog saving her offspring a valuable one. There was a close similarity in the mistakes made by human beings in crossing tracks too frequently. One member remarked that the fact that pure bred animals were more intelligent than mongrels could be attributed to their more frequent association with human beings. The president called to mind that on more than one occasion horses and dogs had, after having undergone treatment at the college, returned when in need of medical aid.

At the next meeting Messrs. Brainerd and Ewing will read papers.

The next meeting of the Society was held on Nov. 28th, the president, Dr. Mills, occupying the chair. There was a very large attendance of members. Dr. Mills communicated the contents of a letter that he had received from a citizen illustrating companionship exhibited by two tramp dogs that for two years past have visited the fountain in Phillip's square. The bigger one of the two is usually in advance of his companion, and when the smaller one lags behind always waits for him to come up. Their attachment to each other is remarkable, and they both resent any interference from other dogs. At night the old one has been found curled up around the smaller one in some doorway.

The essayists of the evening were Messrs. Wylie and Orr. The former gentleman, under the title "The Intelligence of a Bulldog and the Collie," described some interesting incidents that had come to his notice. A bulldog he once owned developed great propensity for hunting, which is rather the exception than the rule with this breed, his favorite sport being the capture of ground hogs. This he did in a clever manner by intercepting the animal on its return to its burrow. By stealthy means he was successful in catching rabbits. He would pounce upon them unawares from behind, thus showing that he realized his success would not depend less on his fleetness than on strategy. A collie he also possessed had developed the "setting" instinct and would retrieve game in a dexterous manner.

Mr. Orr related a most remarkable case of intelligence displayed by a well known trotting mare in this province. She occupied a stall adjacent to that of a cow. Being in training and under special diet, it was noticed she tended to accumulate more flesh than could be accounted for. One evening he discovered her in the act of robbing the cow of her evening meal of grain. She stretched her head over the partition, quietly lifted the pail into her own manger, partook of the contents, and then, to clearly show that she foresaw the results that would have followed had her trick been discovered, placed the empty pail in front of the dejected cow. This act she repeated on every occasion. Some might think she returned it with the expectation of having it refilled, but after a few times she would have discovered that her wish was not gratified. He recounted several instances illustrating memory, revenge, and sense of duty displayed by some of his own animals.

The essays were both of considerable merit and were complimented by the president on the manner of their delivery.

An article on "Instinct," by C. F. Amery, which appeared in a recent edition of *Science*, was than read, at the request of the president, by one of the members. The writer's definition of instinct is that it is due to sensations being transmitted from their several local seats to the brain, where they present themselves as cravings, desires and appetites, imperatively calling for relief Instinct impels to action, but does not guide to its performance.

After a few remarks by some of the members the meeting was then adjourned until after the Christmas vacation, when Messrs. Tracey and Thayer will read papers.

At the meeting held the previous fortnight Mr. McGuire detailed some incidents showing great intelligence in a fox terrier he owned In its mature years it had not failed to remember the whistle Mr. McGuire had taught it to respond to when a puppy, although now the property of another gentleman, and would always show great joy on his return. He knew of a collie that usually accompanied its master when out driving. On one occasion this person having entered a house the horses became frightened and bolted. The dog immediately ran into the house and showed by unmistakable signs, that something was wrong outside. He had seen another dog once drive a chicken back to the yard from which it had wandered ; and a collie belonging to a friend of his would waken the groom of a livery stable by barking whenever any horse returned late at night, thus showing that he knew it was necessary for the animals to be assisted to their respective stalls.

The other essayist, Mr. Brainerd told of an old running mare, the favorite place for contesting her superiority being a level stretch on the road Years after, when she had served well her time, and had long since retired from the turf, the owner's wife had occasion to drive her. All went well until she came to the old course, when, to the surprise and consternation of the old lady she made the distance against time. This showed force of habit. An instance of a collie illustrated the deplorable state of the law with regard to dogs in some of the States of the Union. This dog was in the habit of taking and bringing the cows to and from the pasture. One of the cows having entered the garden, he followed it for the purpose of driving it out. A relentless boy threw a stone, severely hurting him. He was noted for his kindness, never having been known to bite anybody, but a few days later bit the boy. For this he had to pay with his life.

A meeting of the society was held on the 10th of Jan., 1893, the president, Dr. Mills, in the chair. Mr. Stevens read a lengthy and somewhat humurous paper on "The Intelligence of the Cat." He thought the cat was entitled to more study than it usually received, this probably because it does not seek to win attention and affection as does the dog. The nature of the dog is open and demonstrative ; that of the cat is reserved. It is easy to gain the affection of a dog and difficult to lose it On the other hand, it is difficult to win the affection of a cat and easy to lose it. As with all our domestic animals, so with the cat, intelligence can only be developed in proportion to the kindness and good treatment shown towards it. He deprecated the practice of shutting out cats at night time. By this practice for generations certain of them have developed a hereditary habit of night prowling. Treated as they should be, they will soon abandon nocturnal wanderings. However, even in this, cats have individuality, and there are some curious and perverse exceptions. Careful observation goes to show that the cat's native inclination is to hunt the mouse or the rat for sport rather than for food, and a cat that is well cared for is more likely to be successful as a sport seeker than a hungry grimalkin ; firstly, because it is more alert, and, secondly, because it is cleaner. A hungry and unhappy cat does not keep its coat clean, and the keen-nosed mouse can therefore easily sniff out its whereabouts. All cats have not the instinct for mousing. Some, having been deprived of their kittens, have been known to act as foster-mothers to young mice or rats; and not even the pangs of hunger will make a mouser of a cat that has not inherited the instinct of that form of sport—an instinct that seems to run in families—like the taste for fox hunting in human beings.

The other essayist of the evening, Mr. Tracy, read an interesting paper, entitled "How do Animals Reason?" which showed much original scientific thought on the part of the writer. Such a question will often occur to persons of a sympathetic and enquiring nature and not those whose only investigations cease when they have found out the mechanical value of the animal to them. A person would hardly succeed in proving the power animals possess of generating ideas by a mere summary of instances of apparent reasoning. Firstly, a man must be able to account for his own thoughts; secondly, he must have a good knowledge of comparative anatomy and physiology and, above all, be a good observer. If he understands himself and knows enough about the structure and functions of the animal body to know that it is constructed after the same plan as his own, and has observed actions in, say, a dog, which strike him as being calculated and carried out much in the same manner as he himself would carry them out, and then draws his conclusions, his word should be more relied on than the person who listens to stories and attributes all to instinct, or because it is "the nature of the beast" to do so and so. His line of argument went to show that all reasoning phenomena have as their basis reflex nervous impulses, that they are essentially such vital processes, as opposed to automatism.

The next meeting was held on February 1st, the President, Dr. Mills, occupying the chair. The essayists of the evening were Messrs. Plaskett and Thayer. The former gentleman detailed in a comprehensive manner his views on the "Scope of the Animal Mind." The term "mind" is, broadly speaking, correlated with such nervous phenomena as do not come under the sphere of vegetative functions. Some occurrences illustrating intellectual power were quoted. A favorite mare, that had always shown a happy and contented mien, became greatly changed at the time her offspring was taken from her to be weaned. She would mope heedlessly around the pasture and appeared to be engaged in a search for her foal. At the same time the colt seemed equally averse to reconciliation to his solitary fate. On the fourth day they both appeared to have forgotten their sorrows, but the door of the colt's stable was found to be open. A watch was set, when it was found that the mare came about dusk and herself opened the latch with her lips and entered the stable. She remained all night with her offspring, and, as if fully understanding she was violating the decree of her owners, left before dawn and returned to the pasture, admitting herself by opening the gate also. She never became again the same confiding animal she was previously to her painful experience. He thought that the opening of the door and gate showed that animals may learn by imitation to perform certain actions, a view which the President afterwards suggested should not be too strongly advocated as they do not seem to imitate as much as they might. The physiology and pathology of the human brain has been worked out largely by the comparative method, but when the psychology is considered, this does not apply for the following reasons: 1. Impossibility of demonstrating mental processes. 2. Impossibility of man divesting himself of his own psychical loftiness and viewing he actions of his "poor relations" from their own plane. 3. Their inability to speak or write the language of man or the impossibility of man determining the condition of their mind from language, expression, etc. Stalking of game has been correctly likened to the tactics employed by carnivorcus animals in approaching their prey In both cases it has been called instinctive To this he objected, quoting the methodical manner in which wolves approach their prey, which methods have been utilized by the Indians and hunters to successfully come upon deer They excite the curiosity of the quarry in a peculiar manner by making gradually narrowing circles around it until close enough to run it down, sometimes spending half a day in this If actuated by purely instinctive motives they would be morely likely to rush blindly at their distant prey. A tame fox he possessed tunneled the earth as far as its chain would permit it. At the distant opening of one of these holes it placed scraps in order to entice the poultry. This it succeeded in doing on more than one occasion and was not slow to make a meal of any unsuspecting duck that ventured too close. He touched on language, pointing out that this included the suggestion of ideas by other means as well as by voice Tone, perhaps imparted to some animals, especially dogs, the wishes of their masters, as he knew a collie that would only go to fetch the cows if told in an authorative manner and at the right time of day.

The title of Mr. Thayer's paper was "Modified Sex as Affecting Psychic Character." The superiority of the horse as existing in the state of nature to one as altered by man was pointed out, followed by an investigation into the cause thereof from a physiological standpoint He claimed that modification of the sex of the horse did not hurt the animal as an individual as much as it did the dog or other domestic animals, showing how this manifested itself physiologically as well as psychically. The psychological make-up of the entire horse and the modified animal were then contrasted. The attention of the members was then called for a few moments to the female it being suggested by the writer that for physiological reasons non-sexing of the female would probably not affect her as profoundly as the male, and the maternal state as affecting the female mind was also discussed. Maternity was largely instinctive, springing from reflexes, giving as illustrative of his views the case of a bitch without milk having been known to adopt a litter of strange puppies from their plaintive cries evoking past reflexes in that of her nervous system concerned in the function of lactation.

The regular fortnightly meeting of the Society for the Study of Comparative Psychology was held on March 1st, the president, Dr. Mills, in the chair. The essayist of the evening was Mr. Cecil French, and the subject of his essay "The Expression and Communication of Thoughts by Means of Definite Sounds in the Lower Animals." This subject was fast becoming one of great interest among scientific thinkers There should be no question that members of the higher orders are capable of expressing thoughts by definite sounds as evident to all observers. The apparent motives prompting these sounds, the variety of sounds emitted and the extent to which this faculty was developed in the various classes of animals was carefully pointed out; ranging from insect life, the sounds made by the bee when angered, of the beetle or locust to attract the female, among reptiles, the warning hiss of the serpent; among birds the range of tone was enormous, the variety of expression well marked and varied; in the mammalia, the domestic animals and quadrumana received special attention; the neighing of a horse when looking for his food, as well as the variety of expression in the voice of a dog, were wonderful and pointed to certain conclusions which may be proven to be a well defined language. This view was supported by Darwin. Mr. French in his paper gave a short outline of the investigations of Prof. Garnier, who had done some very interesting work in this line on monkeys, and who was at present in Africa continuing these investigations.

On Feb. 16th a paper was read by Mr. Sturrock on "The Psychic Status of the Cat," based on original observations.

At the close of the session a meeting was held for the presentation of diplomas of honorary fellowship and the prizes for the best essays of the year.

To secure the diploma a member must have attended two thirds of all the meetings of the Society during two years, and presented a satisfactory original thesis on some subject in comparative psychology.

The prizes for the best essays were awarded by a special committee and went to Messrs. Thayer 1st, and French 2nd.

